# Supplementary figures and images for: Dual RNA Sequencing Meta-analysis in Plasmodium Infection Identifies Host-Parasite Interactions
Source: mSystems. 2021 Apr 20;6(2):e00182-21. doi: 10.1128/mSystems.00182-21 (PMC8546971; doi:10.1128/mSystems.00182-21)

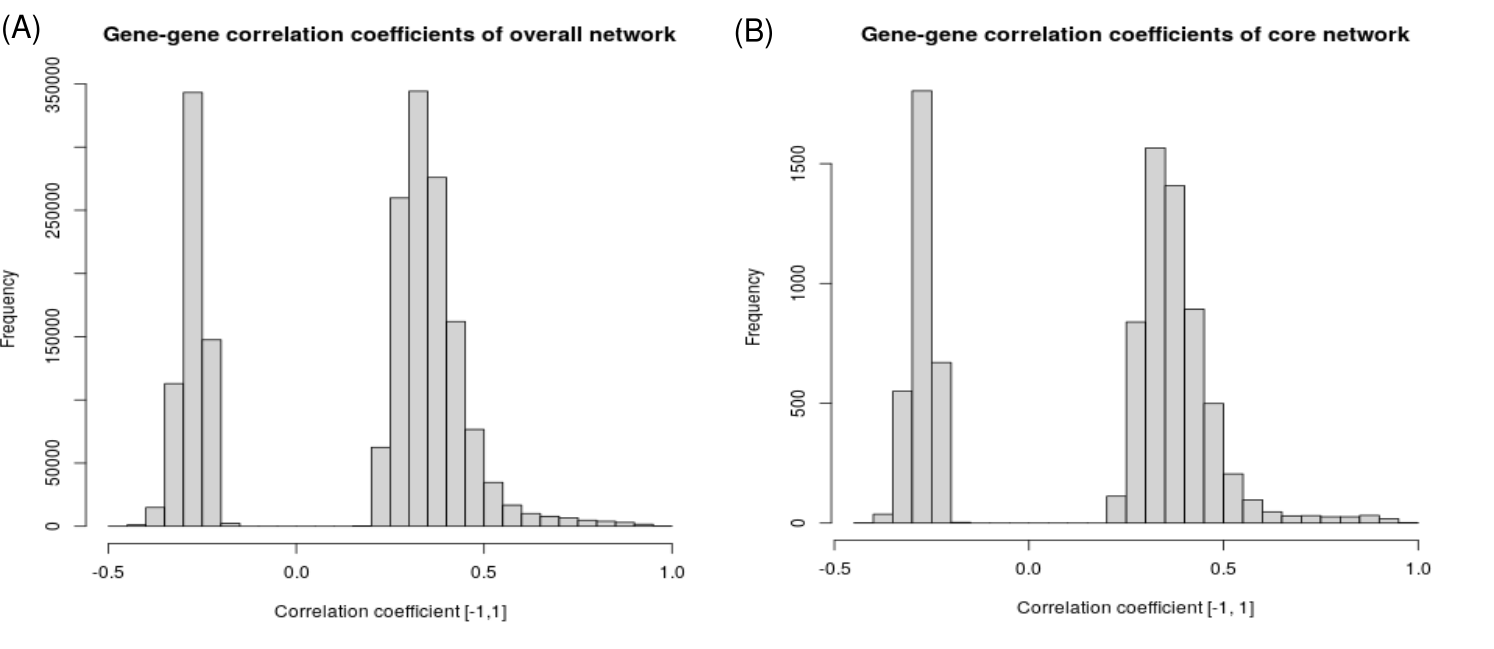
Figure S1.

Supplement: FIG S1 [file msystems.00182-21-sf001.docx]
